# Supplementary material for: Economic evaluation of Health Extension Program packages in Ethiopia
Source: PLoS One. 2021 Feb 2;16(2):e0246207. doi: 10.1371/journal.pone.0246207 (PMC7853458; doi:10.1371/journal.pone.0246207)
Supplement: Attachment — Submitted filename: Reviewer response letter, Dec 13.docx [file pone.0246207.s002.docx]

Dec 16, 2020

To: Athina Economou and/or other editor(s):

Journal: PLOS ONE

Dear Editors,

Thank you for allowing us to submit a revision of our paper “Economic evaluation of Health Extension Program packages in Ethiopia” (PONE-D-20-27778). We thank the reviewers for their constructive suggestions and valuable comments. A point-by-point response to the reviewer’s and editors comments can be found below. All page numbers apply to the “Main document clean version” document.

Thank you very much for your consideration of our manuscript. We look forward to hearing from you again.

Best wishes,

Lelisa Fekadu on behalf of all authors

**Reviewer 1**

1. The manuscript is well written and easy to follow. However, considering the following will improve the manuscript: In the introduction, you mention that the country has made substantial improvements in health outcomes. Provide a brief synopsis of the statistics to show those improvements.

*Reply: Thank you for the comment and we added the sentence in page 4 and line number 88-92. “Ethiopia has made substantial improvements in health outcomes: for example, the maternal mortality ratio (MMR) decreased from 676 deaths per 100,000 in 2011 to 401 in 2017. Under-five mortality per 1,000 live births decreased from 123 in 2005 to 59 in 2019 leading to an average life expectancy at birth of 65.5 years. HIV, Tuberculosis (TB), and malaria-related morbidity and mortality have significantly decreased in the last decades”.*

**Reviewer 2**

2. The manuscript is technically sound except for a few issues. In your costing approach, there was no mention of the possibility of double counting costs using the two approaches and how you handled double counting if it happened. If no double counting happened please mention it.

*Reply: This is an important point, and we fully agree when combining the result of the two methods there is a possibility of double counting of certain resources. However, in this particular analysis, we have ensured that these resources are cross-checked and accounted for at one location in order to avoid double counting. Therefore, we added the sentence in page number 7 and line number 131-132 “All cost inputs from both methods were crosschecked and accounted for to avoid double-counting”.*

3. Please explain why the 2018 inflationary and exchange rate was used for costs data collected in 2019.

*Reply: Thank you. We need to clarify that even though the primary data collection takes place between 7 June 2019 and 1 July 2019, real cost data has been collected for the year 2018 and before that date, which justifies the use of 2018 average annual exchange rate.*

4. In table 2, please align supervision and its description properly.

*Reply: Thank you. The cost inputs and its description is now aligned in Table 2.*

5. Please indicate what software was used to make the linear interpolation between baseline and current coverage before the Spectrum analysis.

*Reply: Thank you for your comment. We need to clarify that the spectrum software ((live saved tool -LiST, FamPlan, and TB impact and modelling estimate-TIME) is used to interpolate between the baseline and current coverage as indicated in page 9 line 170-174.*

6. Personnel costs contribution for hygiene and environment has 3 percentage points but 4 items mentioned; utilization of latrines seems to have no percentage point mentioned

*Reply: We appreciate the reviewer’s comment and agree that we have missed one-percentage points. The sentences on page 14 and line number 263-266 have now been revised as “Personnel costs accounted for 18%, 24%, 24%, and 32% of the costs of improved water sources, hand washing with soap, the hygienic disposal of children’s stools, and the use of latrines, respectively. The remaining share of the expenditure was contributed by capacity building, including start-up and monitoring and evaluation.”*

7. Major part of the discussion was comparing study results with other studies and little on impact of these results on current policy.

*Reply: We appreciate the reviewer’s comments and agree that the discussion section needs to be strengthened by the impact of the result on the current policy. Thus, we have added the following two sentences on page 17 and line number 331-334.*

*The sentence now reads “The unit costs and cost-effectiveness of the HEP based on health need and supply constraints provide contextual evidence for decision-makers to prioritize healthcare interventions and allocate resources efficiently in order to improve population health.” And in page 18 and line number 364-366 “Thus, the HEP leads to a higher proportion of lives saved and a more cost-effective approach to delivering essential health services to rural and vulnerable communities, where access to qualified staff is limited”.*

8. Small grammatical errors throughout the manuscript that need revision.

*Reply: Thank you. We had attempted to correct the grammatical issues accordingly.*

**Response to comments from academic editor**

9. Please ensure that your manuscript meets PLOS ONE's style requirements, including those for file naming.

*Reply: Thank you for your comment. This manuscript has been revised according to the journal style requirements (please see the revised manuscript).*

10. We note that you have indicated that data from this study are available upon request. PLOS only allows data to be available upon request if there are legal or ethical restrictions on sharing data publicly.

*Reply: Thank you for the suggestion. The data set used for this study has been uploaded as supporting information (in the revised manuscript).*

11. Please amend your list of authors on the manuscript to ensure that each author is linked to an affiliation. Authors’ affiliations should reflect the institution where the work was done (if authors moved subsequently, you can also list the new affiliation stating “current affiliation:….” as necessary).

*Reply: Thank you. We have amended the list of authors and ensured that each author is linked to an affiliation.*

12. Thank you for stating the following in the Financial Disclosure section: 'Bill & Melinda Gates Foundation funded the study. The funder had no role in the conception, data collection, analysis and write up of this study.' We note that one or more of the authors are employed by a commercial company: Abenezer Consulting and Research PLC, and Monitoring, Evaluation, Research and Quality Improvement (MERQ) consultancy.

Please provide an amended Funding Statement declaring this commercial affiliation, as well as a statement regarding the Role of Funders in your study. If the funding organization did not play a role in the study design, data collection and analysis, decision to publish, or preparation of the manuscript and only provided financial support in the form of authors' salaries and/or research materials, please review your statements relating to the author contributions, and ensure you have specifically and accurately indicated the role(s) that these authors had in your study. You can update author roles in the Author Contributions section of the online submission form.

Please also include the following statement within your amended Funding Statement.

“The funder provided support in the form of salaries for authors [insert relevant initials], but did not have any additional role in the study design, data collection and analysis, decision to publish, or preparation of the manuscript. The specific roles of these authors are articulated in the ‘author contributions’ section.”

If your commercial affiliation did play a role in your study, please state and explain this role within your updated Funding Statement.

*Reply: Thank you for the clarification and suggestions. We have updated these sections accordingly. Please see pages 22 and 23, line number 471-478.*

13. Please also provide an updated Competing Interests Statement declaring this commercial affiliation along with any other relevant declarations relating to employment, consultancy, patents, products in development, or marketed products, etc.

Within your Competing Interests Statement, please confirm that this commercial affiliation does not alter your adherence to all PLOS ONE policies on sharing data and materials by including the following statement: "This does not alter our adherence to  PLOS ONE policies on sharing data and materials.” (as detailed online in our guide for authors http://journals.plos.org/plosone/s/competing-interests) . If this adherence statement is not accurate and  there are restrictions on sharing of data and/or materials, please state these. Please note that we cannot proceed with consideration of your article until this information has been declared.

Please include both an updated Funding Statement and Competing Interests Statement in your cover letter. We will change the online submission form on your behalf. Please know it is PLOS ONE policy for corresponding authors to declare, on behalf of all authors, all potential competing interests for the purposes of transparency. PLOS defines a competing interest as anything that interferes with, or could reasonably be perceived as interfering with, the full and objective presentation, peer review, editorial decision-making, or publication of research or non-research articles submitted to one of the journals. Competing interests can be financial or non-financial, professional, or personal. Competing interests can arise in relationship to an organization or another person. Please follow this link to our website for more details on competing interests: http://journals.plos.org/plosone/s/competing-interests

*Reply: Thank you for the clarification and suggestions. We have updated these sections accordingly. Please see page 22, line number 467-469.*

14. We note you have included a table to which you do not refer in the text of your manuscript. Please ensure that you refer to Table 2 and 4 in your text; if accepted, production will need this reference to link the reader to the Table.

*Reply: We have now referred Table 2 and 4 in the main text of the revised manuscript.*
